# Exploring women’s experiences participating in yoga after a cancer diagnosis: a protocol for a meta-synthesis

**DOI:** 10.1186/s13643-022-02042-y

**Published:** 2022-08-12

**Authors:** Jenson Price, Jennifer Brunet

**Affiliations:** 1 School of Human Kinetics, University of Ottawa, Ottawa, Ontario K1N 6N5 Canada; 2 Cancer Therapeutic Program, Ottawa Hospital Research Institute, The Ottawa Hospital, Ottawa, Ontario Canada; 3 Institut du savoir Montfort, Hôpital Montfort, Ottawa, Ontario Canada

**Keywords:** Qualitative, Oncology, Yoga, Women

## Abstract

**Background:**

The benefits of yoga for clinical and non-clinical populations have been summarized in published systematic reviews. The vast majority of systematic reviews on the topic are syntheses of quantitative research that evaluated the effects of yoga. As qualitative research related to women’s experiences participating in yoga after a cancer diagnosis is growing in quantity, systematic synthesis and integration of qualitative research are necessary to facilitate the transfer of knowledge. This paper describes the protocol for a meta-synthesis of qualitative research exploring women’s experiences participating in yoga after a cancer diagnosis.

**Methods:**

Using a meta-study methodology, six electronic databases were searched to identify relevant articles. Additionally, the reference lists of relevant articles retrieved during the electronic database search were scanned to identify other relevant articles. Two reviewers independently screened the titles and abstracts, retaining those that appeared to relate to the review objectives. Next, they reviewed the retained full-text articles to assess eligibility according to four inclusion criteria. They will extract data from eligible studies and assess the quality of included studies. Data analysis will involve three main analytical steps: meta-data analysis, meta-method analysis, and meta-theory analysis. Findings from the three analytical steps will be interpreted collectively to generate additional insights beyond the findings of the primary studies to facilitate a more comprehensive understanding of women’s experiences participating in yoga after a cancer diagnosis.

**Discussion:**

By systematically collecting, analysing, and interpreting findings across multiple primary qualitative studies, we will develop an overarching narrative and interpretation of the role and value of yoga for women diagnosed with cancer. A synthesis of qualitative research is vital as it embraces the heterogeneity of the research so as to provide important context for understanding the experiences of various women participating in yoga.

**Systematic review registration:**

PROSPERO CRD42021229253

**Supplementary Information:**

The online version contains supplementary material available at 10.1186/s13643-022-02042-y.

## Background

Globally, more than eight million women are diagnosed with cancer each year [1]. The cancer burden continues to grow, exerting tremendous physical, emotional, and financial strain on individuals, families, communities, and health systems [2, 3]. Survival rates for many types of cancers have improved in high-income countries due to accessible early detection tests, improved treatment, and better survivorship care. In Canada, the predicted 5-year net survival rate for all cancers combined is 65% for women [4]. However, approximately one in three women who complete treatment for cancer report acute and chronic adverse effects that may be visible (e.g. scarring, disfigurement, physical deconditioning, hair loss [5, 6]) or nonvisible (e.g. hot flashes, nausea [7, 8]), which can have a lasting impact on their overall wellbeing and quality of life [9, 10]. Moreover, women often report significant alterations to their thoughts, feelings, and behaviours towards their body, self, and others [8, 11–14], which can also impair their wellbeing and quality of life [15–19]. There is a need to identify strategies to help support women’s wellbeing and quality of life after they receive a cancer diagnosis.

Yoga is classified by the National Institutes of Health as a form of complementary and alternative medicine [20]. Investigation of the benefits of yoga for individuals diagnosed with cancer began in the 1990s and took off in the following decade. There is now evidence that yoga can effectively promote wellbeing and quality of life through its positive influence on physical (e.g. decreases fatigue, increases physical functioning) and psychological (e.g. reduces anxiety and depression) health outcomes during and after cancer treatment [21–25]. Additionally, there is evidence that participating in pleasurable activities that encourage a mind-body connection, such as yoga, can foster positive thoughts, feelings, and behaviours towards the body, self, and others after a traumatic experience (e.g. cancer [5, 6]).

The use of qualitative research is proliferating in the area of sport and exercise psychology [26] as qualitative methods (e.g. interviews, focus groups, open-ended survey questions) are valuable means to gather knowledge that may not be accessible using methods relied upon in quantitative research (e.g. surveys with close-ended questions). Qualitative research provides detailed explanations and meanings given by the individuals who take part in an activity (e.g. yoga). Consequently, it can offer a thick description of a phenomenon, which is when researchers are able to delve deeper into the nature of a phenomenon by considering contextual information when observing and interpreting participants’ explanations and meanings [27]. Thick description thus not only provides a description of a phenomenon under investigation but also attempts to document the complexity and multiplicity of individuals’ experiences and meanings [28]. Although valuable, primary qualitative studies are rarely used on their own to contribute to practical knowledge [29]. To enable qualitative research to contribute to practical knowledge, a systematic review and integration of findings from the collective body of qualitative research is critical. Yet, most published systematic reviews on yoga present syntheses of quantitative research that evaluate the effects of yoga. Moreover, these reviews have generally focused on specific outcomes. For example, O’Neill et al. [30] focused on the effects of yoga on cancer-related fatigue and quality of life. Whilst such reviews address essential questions about the magnitude of effects for specific outcomes, they are subject to limitations. Most notably, the statistical combination of findings from multiple independent studies to obtain an overall statistic that summarizes the effect of an experimental intervention (e.g. yoga) on an outcome (e.g. quality of life) can mask important differences within and across studies [31, 32] and thus can be misleading. In addition, when data are sparse (e.g. small sample sizes in primary articles, small number of included articles), the estimate effect size may be biased. Moreover, quantitative evidence syntheses do not necessarily include all available evidence that may provide insight into the benefits of a certain activity; there are many published studies where authors have used qualitative methods to gather such insight that are not included in reviews. Although these qualitative data cannot be expressed numerically, there is concern with their exclusion because qualitative research plays an important role in deepening our understanding of the context for which these interventions are conducted [29]. This is one of the key motivations for doing qualitative evidence synthesis.

Qualitative evidence synthesis is a valuable tool for understanding the experiences of individuals who take part in an activity, both comprehensively (due to the qualitative approach) and broadly (due to the integration of studies from different contexts and with diverse participants), so as to draw conclusions about the benefits of an activity (e.g. yoga) based on several studies. Qualitative evidence synthesis is especially important considering variations across studies in terms of the participants, interventions, theoretical perspectives, study design, and outcome measurement tools. This variability, often described as clinical diversity and methodological diversity, produces highly heterogenous data that can be misrepresented in quantitative evidence syntheses [33]. However, heterogeneity arising from the clinical and/or methodological diversity across studies is welcomed in qualitative syntheses, whereby it is appropriate to review, analyse, and synthesize studies with diverse clinical and methodological factors to provide a meaningful summary of participants’ perspectives on the role and value of an activity. The use of qualitative evidence synthesis to explore the explanations and meanings given by individuals participating in different activities, including yoga, is growing. For example, Capon et al. [34] reviewed 11 articles reporting on studies investigating the lived experiences of people with mental health conditions who participated in yoga for mental health outcomes (e.g. post-traumatic stress disorder, depression, anxiety). Despite clinical diversity across the primary qualitative articles reviewed, Capon et al. [34] formulated three overarching themes, namely self as an agent of change, alleviation of suffering, and healing as a process. Their findings provide necessary insight into the process of healing for people with mental health conditions as well the potential mechanisms underlying this process to offer suggestions for clinical practice. Nevertheless, there remains a gap in the synthesis of qualitative evidence on women’s experiences participating in yoga after a cancer diagnosis.

### Current study

In spite of a proliferation of primary qualitative articles on women’s experiences participating in yoga after a cancer diagnosis, an analysis, synthesis, and interpretation of the collective findings are lacking. Capitalizing on the potential of qualitative research is necessary to provide a meaningful summary of participants’ experiences to develop theory and provide direction for practice or policy development. Thus, the aim of this paper is to describe the protocol for a meta-synthesis using a meta-study methodology to analyse, synthesize, and interpret qualitative evidence from research on the experiences of women who have participated in yoga after a cancer diagnosis to generate an understanding of their experiences. The specific research objectives are as follows: (1) integrate findings from primary qualitative articles exploring the accounts of women who participated in yoga after a cancer diagnosis, (2) compare and contrast the findings to elucidate patterns or contradictions in conclusions, and (3) develop an overarching narrative of women’s experiences participating in yoga after a cancer diagnosis.

## Methods

### Conducting a qualitative evidence synthesis

Qualitative evidence synthesis, described as narrative synthesis, qualitative synthesis, meta-ethnography, or meta-synthesis, refers to methods used to synthesize a number of qualitative research studies within a particular field of study [35]. As with methods used to conduct quantitative evidence synthesis, qualitative evidence synthesis methods use a staged approach (e.g. identify, screen, appraise quality) to analyse and synthesize relevant articles. For this review, a meta-synthesis using a meta-study methodology [29] is appropriate because there is a need to analyse the findings, methods, and theory used in primary qualitative research before a complete synthesis is conducted to generate new understandings and theories in this evolving field of research. Meta-study entails two distinct phases: (1) analysis and (2) synthesis. There are three types of analysis: meta-data analysis, meta-method analysis, and meta-theory analysis. Findings from the included articles will be coded to identify patterns and themes in the data and then interpreted to provide additional insights, beyond what is offered in the primary studies, into the phenomenon from the point of view of women diagnosed with cancer who participated in yoga.

### Procedures and analysis

To complete this meta-study meta-synthesis, recommendations outlined by Paterson et al. [29] will be used. Their recommendations are organized into six distinct but overlapping steps: (1) formulating a research question, (2) selection and appraisal of the primary research, (3) meta-data analysis, (4) meta-method analysis, (5) meta-theory analysis, and (6) meta-synthesis. Steps 3 to 5 do not necessarily unfold sequentially and are frequently conducted concurrently. The details of these six steps are described below, and reporting herein follows the Preferred Reporting Items for Systematic review and Meta-Analysis Protocol (PRISMA-P [36]) guidelines (see Supplemental file 1 for checklist). The protocol for this review has been registered on PROSPERO (registration number: CRD42021229253). The Preferred Reporting Items for Systematic review and Meta-Analysis (PRISMA [37]) guidelines will be followed when reporting the results from this review to ensure complete and transparent reporting.

#### Step 1: Formulating the research questions

Before conducting the literature search, the purpose of this review and the specific research questions were established, leading to the clarification of the inclusion criteria. The specific research questions are as follows: (1) what are the experiences of women who have participated in yoga after a cancer diagnosis, (2) what elements of yoga contribute to participants’ positive or negative experiences, and (3) what are participants’ barriers, motives, and preferences for their participation in yoga interventions and programmes? In addition to answering these research questions, the review will also aim to describe knowledge gaps and make recommendations for future research.

#### Step 2: Selection and appraisal of the primary research^1^

##### Step 2a: Identifying relevant articles

Articles were retrieved by searching six electronic databases: Medical Literature Analysis and Retrieval System Online (MEDLINE), Cumulative Index to Nursing and Allied Health Literature (CINAHL), PsycINFO, Scopus, SPORTDiscus, and Web of Science. With the help of a university librarian, a sensitive search strategy was developed drawing on Medical Subject Heading (MeSH) terms and keywords that have been used in published reviews (e.g. [38]). MeSH terms and keywords used covered the population (i.e. women diagnosed with cancer) and terminology associated with yoga (e.g. yoga, mindfulness, breathing exercises, meditation). No MeSH terms and keywords covering methods (e.g. interviews, focus groups) were used, as these have undergone little validation [39], and qualitative articles have been known to produce poor descriptors of the research methods, with neither the title nor abstract explicitly stating the methods used [40, 41]. The search strategy was pilot tested and finalized in MEDLINE (see Table 1 in Appendix for the final MEDLINE search strategy) before being translated for use in the five other databases. This search for articles from database inception onwards was completed in November 2020, and results were exported into Covidence software [42] for automatic removal of duplicates. In August 2021, this search was supplemented by scanning the reference lists of relevant articles retrieved during the electronic database search (i.e. reviews, included studies) to ensure all pertinent studies were identified. The database search was replicated in October 2021 to retrieve citations published during the previous 11 months. Covidence software was used to store, organize, and manage all the references.

The inclusion criteria emerged directly from the research questions guiding this meta-synthesis and were set a priori. The inclusion criteria are as follows: (1) primary studies conducted with adult women (≥ 18 years) diagnosed with cancer, regardless of type of cancer, stage of the disease, and phase on the cancer continuum (e.g. diagnosis, treatment, post treatment, palliation), (2) used qualitative methods to collect data (e.g. interviews, focus groups, observations, journaling, open-ended survey questions), (3) report on participants’ experiences engaging in yoga of any type and intensity, and (4) were original research published in English language in a peer-reviewed journal. No restriction was placed on year of publication and study design (i.e. observational or experimental). The exclusion criteria are as follows: (1) the sample is composed of > 50% men diagnosed with cancer (to ensure that the sampling method does not introduce bias in the analysis because excluding articles that have > 50% women could result in a biased sample as not all women would have equal chance to have their experiences considered in the synthesis), (2) qualitative findings are not presented, (3) do not report on participants’ experiences engaging in yoga, and (4) abstracts from conference proceedings, unpublished theses, books, and reviews. Finally, no restriction was imposed for type of cancer to allow for breadth of experiences to be captured in the synthesis.

##### Step 2b: Study selection

After the removal of duplicate records, two reviewers independently screened the titles and abstracts. They retained those that appeared to relate to the eligibility criteria and tracked reasons for exclusion otherwise. They retained records of titles and abstracts that were vague for full-text review. Next, the same two reviewers reviewed the full-text of remaining records to assess eligibility according to the eligibility criteria and tracked reasons for exclusion. At both steps, a third reviewer made the final decision when disagreements arose. Cohen’s kappa, a statistic reflecting level of agreement among independent people assessing qualitative data, will be calculated for both screening steps to report on inter-reviewer reliability and presented with the results. For this review, Cohen’s kappa was chosen as the marker for reliability as it accounts for the possibility of agreement occurring by chance and thus is generally considered a more robust measure of reliability than simple percent agreement calculation [43]. Coefficients will be interpreted as follows: 0–0.20 = none, 0.21–0.39 = minimal, 0.40–0.59 = weak, 0.60–0.79 = moderate, 0.80–0.90 = strong, and > 0.90 = excellent agreement [43]. A PRISMA diagram of the search results and reasons for exclusion will be prepared and presented alongside the results.

##### Step 2c: Data extraction

Data extraction will be conducted by two independent reviewers using a template for collecting data housed on Covidence. After uploading the full texts to Covidence, both reviewers will independently extract the following information from each eligible article: authors, country where data were collected, year of publication, study objective(s), sample characteristics (i.e. age, percent female/women), sampling method(s), sample size, type of cancer(s), disease stage(s), phase(s) on the cancer continuum, reported yoga characteristics (i.e. length, duration, frequency, location(s) of practice, style, group or individual), methodology (e.g. constructivist, interpretivist), data collection methods (e.g. semi-structured interview, focus group), analysis methods (e.g. grounded theory, interpretative phenomenological analysis, thematic analysis, content analysis), conceptual/theoretical approaches, and key qualitative findings. In studies that used an experimental design, data on the trial will also be extracted. If the information presented in the original article is unclear or missing, the corresponding author of the article will be contacted via email for clarification (maximum two attempts).

##### Step 2d: Quality assessment

Although there is no consensus about whether quality criteria should be applied to qualitative research, a growing number of researchers are choosing to appraise qualitative studies in meta-syntheses [44]. Included studies will be appraised using the Consolidated Criteria for Reporting Qualitative Research (COREQ) 32-item checklist [45]. The checklist consists of items specific to the research team and reflexivity (i.e. personal characteristics, relationship with participants), study methods and context (i.e. theoretical framework, participant selection, setting, data collection), analysis, findings, and interpretations. As there is no empirical basis to rate the quality of reporting of qualitative research, studies will not be excluded from analysis on the basis of the COREQ results; rather, the intention is to allow readers to evaluate the quality of reporting of studies conducted thus far based on our appraisal results [44]. Therefore, the results of the COREQ checklist assessment will be narratively presented for all studies included in the synthesis [44].

#### Step 3: Meta-data analysis

The first analytical step involves an analysis of the findings presented in the primary qualitative articles. Thematic synthesis, which draws on methods of thematic analysis for primary qualitative research, is a common approach to qualitative evidence synthesis in health and related disciplines [46]. Thematic synthesis involves three steps. First, extracted text reflecting findings from the included articles will be coded line by line to search for patterns and themes in the data within and across included articles. Second, similarities will be grouped together into themes, and a narrative summary of the results describing the themes will be written. Finally, these themes will be interpreted to explore the implications for understanding the phenomenon. The thematic synthesis will be carried out by two reviewers, and disagreements in coding will be resolved through discussion with a third reviewer; initial codes and themes will be revisited or refined accordingly.

During the meta-data analysis, themes will be entered as columns into a table, and coded data from each article will illustrate the themes in rows to facilitate comparison within and across articles. The aim of this table is to demonstrate themes with illustrative data and capture similarities and differences within the data where possible—that is, to show how themes are similar but also show divergence of findings in each theme, where it applies. This table also serves as a transparent link between the reviewers’ analysis and interpretation and the findings of the primary articles reviewed.

#### Step 4: Meta-method analysis

The second analytical step involves an analysis of the methods used in the primary qualitative articles. This step determines how the interpretation and implementation of qualitative research methods have shaped the research findings and the emergent ideas in this area of research [29]. Meta-method analysis involves four steps. First, data on the methods extracted from the included articles during step 2c will be entered as columns into a table with primary qualitative articles as the rows to allow for comparison between articles, as well as to allow for assessment of coherence of methods within studies. Second, the methods used will be analysed to determine frequency of use and any potential patterns of use. Third, the results from the COREQ checklist assessment from step 2d will be discussed to furnish conclusions about the appropriateness of methods used in the primary studies. Finally, these findings will be analysed to determine whether the use of certain methods has changed over time, drawing attention to the impact of these changes on how researchers have designed their research, and ultimately on the findings reported in the primary qualitative articles.

#### Step 5: Meta-theory analysis

The third analytical step is the analysis of the conceptual/theoretical approaches used in the primary qualitative articles. A meta-theory analysis is an important component of a meta-synthesis because the subtle differences between theories within the same field of scholarship can make significant differences in what is studied, how it is studied, and why it is studied [29]. Extracted theories from the articles reviewed will be examined to identify any major paradigm(s) or ontological approach(es) that may have informed the selection of said theories. The findings will be interpreted to explore any limitations, strengths, or ambiguities that may be influencing the use of theory and interpretation of findings in the primary qualitative articles exploring women’s experiences participating in yoga after a cancer diagnosis.

#### Step 6: Meta-synthesis

In this last step, the results from the proceeding steps will be aggregated to create an overarching narrative of women’s experiences participating in yoga after a cancer diagnosis. Specifically, the goal will be to “go beyond” the findings of the primary studies identified in the previous three steps and generate additional concepts, understandings, and hypotheses that answer the research questions. Furthermore, there will be a deliberate attempt to identify possible contradictions in conclusions across studies. Finally, if necessary, the reviewers will propose alternative conceptual and theoretical structures within which existing knowledge can be interpreted. To achieve this, the meta-data analysis findings will be explored in the context of the meta-method and meta-theory results as follows: (1) identify the nuances in the various methods that have been applied and the patterns in how and why such approaches may have been used, (2) consider the kinds of knowledge that may not have been included in current conceptualizations as a result of methodological choices, (3) understand why the authors of primary research may have reported different findings at different times, and (4) explore new conceptual/theoretical alternatives that might account for a more comprehensive, accurate or credible interpretation of women’s experiences participating in yoga after a cancer diagnosis.

### Rigour

Several strategies will be used to enhance the quality and rigour of this meta-synthesis [47]. First, the findings will be arrived at through triangulation. Specifically, multiple reviewers with different backgrounds and experiences will bring different perspectives to the analysis and synthesis of the primary qualitive findings. Similarly, there are no restrictions placed on the methods, methodologies, epistemological and ontological stances, or theoretical frameworks used in the primary qualitative articles, thus allowing for a breadth of perspectives to be presented and analysed. Second, an expansive and exhaustive search was conducted across several databases to help ensure all relevant published articles were identified. This is in keeping with the goal to saturate findings and to ensure a broad interpretation of the research topic. Third, an audit trail is being maintained throughout the process to keep an accurate and detailed report on retrieval, tracking, and selection. Similarly, memo writing will be used in the classification, analysis, and synthesis of findings and decisions made, whilst direct quotations from the primary qualitative articles will be presented to support conclusions.

## Discussion

Quantitative evidence syntheses have provided data on the benefits and outcomes of yoga for adults diagnosed with cancer [21–25]. Beyond the fact that they omit key findings from the literature by focusing mostly on randomized controlled trials and relying on the aggregation of studies with similar parameters to draw conclusions [31, 32], they face challenges because there is wide clinical and methodological diversity (i.e. there is variability in contexts and populations, a lack of standardized approach in conducting yoga interventions, a multiplicity of outcome measures, and varying doses of interventions (e.g. frequency, length, duration)). Such diversity gives rise to heterogeneity, which is a troublesome aspect of many quantitative evidence syntheses as it might influence the conclusions of the synthesis [31, 32]. However, the epistemological and methodological considerations of qualitative studies lend themselves well to embracing heterogeneity in an evidence synthesis. Therefore, qualitative evidence syntheses represent a valuable approach to synthesize research in this evolving field to continue to drive forward important questions and future endeavours, as well as practice. The current manuscript describes the protocol for a meta-synthesis using a meta-study methodology to analyse, synthesize, and interpret qualitative research on the experiences of women who have participated in yoga after a cancer diagnosis.

Systematic reviews are lauded as the best evidence for answering health research questions, particularly those about intervention effectiveness [37]. This is because they use prespecified methods and analyses that allow for a rigorous, methodical approach to synthesizing research evidence. Yet, concerns have been raised that it can be difficult to discern whether decisions made during the review process are arbitrary, or that the decision to include/exclude studies/data in a review was made in light of knowledge about individual study findings [36]. Consequently, there have been calls to provide detailed, concise, and transparent descriptions of the steps used for data collection and analysis [36]. This is especially important for qualitative evidence synthesis as there are several approaches (e.g. narrative synthesis, qualitative synthesis, meta-ethnography, meta-synthesis) that use different methods for aggregating, synthesizing, and reporting data. Thus, it is necessary to describe the steps being used in this review, which is the objective of this manuscript.

There are several strengths associated with the procedures detailed herein. Multiple gold standard guidelines were followed in the preparation of the review protocol and will be adhered to when reporting the results. By incorporating elements from different guidelines, a solid framework and structure was created by which the research questions can be answered. Second, rigorous and systematic methods will be used, which will ensure a broad range of concepts are captured and will allow for replication of the review. For example, a systematic and explicit search strategy, along with COREQ checklist assessment of the included articles, in line with standard qualitative practice [44, 48], will be used. As well, a university librarian with experience conducting meta-syntheses assisted with database selection and reviewed the search strategy. Third, whilst generalisability is not a goal of qualitative research, meta-syntheses give a depth of understanding across more diverse populations, thereby providing a wider reach than could be achieved in a primary qualitative study. Indeed, by not limiting to a specific cancer type and phase on the cancer continuum, the findings from this meta-synthesis may be relevant to the wider population of women diagnosed with cancer. Finally, the synthesis of primary qualitative studies will allow the voice of women to guide future research decisions around women’s needs, gauge the suitability of interventions, and appreciate the implications of yoga for women after a cancer diagnosis.

Notwithstanding the strengths, there are three key challenges associated with conducting a meta-synthesis using a meta-study methodology. First, like many qualitative evidence syntheses, meta-study relies on decontextualizing concepts to attain greater generalizability [29, 47]. This is at odds with many of the epistemological approaches often employed in qualitative work, which stress the importance of context; however, the value of a meta-study lies in its ability to understand the broader pattern of data in a field by interpreting its varying contexts. Moreover, similar to primary qualitative studies, readers can apply findings to their own research contexts, as they see fit. Second, the quality of the meta-study is heavily reliant on the reviewers' ability to articulate and further interpret the findings of primary studies. As a result, the forthcoming themes and subthemes developed may be different from those developed by other reviewers. Nonetheless, the inclusion of multiple independent individuals involved in the collection, analysis, synthesis, and interpretation of the qualitative findings will ensure multiple perspectives, backgrounds, and epistemological stances are considered during the construction of the final themes and subthemes. Finally, although the appropriateness of assessing the quality of studies in qualitative evidence syntheses is debated, an increasing number of reviewers are performing critical appraisals [44, 49], shifting the discussion in the literature from ‘should we’ to ‘how can we’ appropriately evaluate (and improve) the quality of reporting in primary qualitative studies [44]. Thus, since the focus of a meta-study meta-synthesis is to analyse the methods and theories involved in interpreting the data of primary qualitative studies, the COREQ checklist [45], which evaluates the explicit and comprehensive reporting of qualitative studies, was chosen to appraise articles reviewed.

In conclusion, there is a need to collate the perspectives of women who have participated in yoga after a cancer diagnosis explored via qualitative research. To ensure that all the important decisions and steps taken related to the conduct of this meta-synthesis are reported with transparency and sufficient detail, this manuscript presents in detail the protocol for a meta-synthesis aimed at exploring women’s experiences participating in yoga after a cancer diagnosis. Additionally, this will improve trustworthiness of this review. Publication of the protocol, along with registration on PROSPERO, will also help to avoid wasted research effort by preventing duplication in case other researchers want to conduct a similar review.

### Supplementary Information

**Additional file 1.** PRISMA-P checklist.

## Data Availability

Not applicable

## Appendix

**Table 1:** MEDLINE search strategy

1	(wom?n or female*).ti,ab.
2	Women/
3	Female/
4	1 or 2 or 3
5	exp breathing exercises/
6	exp yoga/
7	exp relaxation/ or relaxation therapy
8	exp meditation/
9	exp mindfulness/
10	(yog* or asana or pranayama or dhyana or meditation or relaxation or mindful*).ti,ab.
11	(breath* adj3 exercise?).ti,ab.
12	(body adj3 posture?).ti,ab.
13	(deep* adj3 breath*).ti,ab.
14	(breath* adj3 technique?).ti,ab.
15	5 or 6 or 7 or 8 or 9 or 10 or 11 or 12 or 13 or 14
16	exp Neoplasms/
17	exp Medical Oncology/ or exp Psycho-Oncology/
18	(neoplas* or oncolog* or cancer* or tumo?r or leuk?emia* or carcinoma* or adeno-carcinoma* or lymphoma* or malignan* or melanoma* or metasta* or sarcoma* or adenoma* or adenocarcinoma* or blastoma* or mesothelioma*).ti,ab.
19	16 or 17 or 18
20	4 and 15 and 19
21	Limit 20 to (English language and humans)

## Abbreviations

PRISMA-P: Preferred Reporting Items for Systematic review and Meta-Analysis Protocols
PRISMA: Preferred Reporting Items for Systematic review and Meta-Analysis
ENTREQ: Enhancing transparency in reporting the synthesis of qualitative research
MEDLINE: Medical Literature Analysis and Retrieval System Online
CINAHL: Cumulative Index to Nursing and Allied Health Literature
MeSH: Medical Subject Heading
COREQ: Consolidated criteria for reporting qualitative research

## Footnotes

1 In line with submission requirements, screening and data extraction were not completed prior to submission (January 21, 2021). However, screening was completed during the review process (January 2021-May 2022); this is reflected in the verb tense of the following section.
